# The circulating soluble form of the CD40 costimulatory immune checkpoint receptor and liver metastasis risk in rectal cancer

**DOI:** 10.1038/s41416-021-01377-y

**Published:** 2021-04-09

**Authors:** Sebastian Meltzer, Annette Torgunrud, Hanna Abrahamsson, Arne Mide Solbakken, Kjersti Flatmark, Svein Dueland, Kine Mari Bakke, Paula Anna Bousquet, Anne Negård, Christin Johansen, Lars Gustav Lyckander, Finn Ole Larsen, Jakob Vasehus Schou, Kathrine Røe Redalen, Anne Hansen Ree

**Affiliations:** 1 Department of Oncology, Akershus University Hospital, Lørenskog, Norway; 2 Department of Clinical Molecular Biology, Akershus University Hospital, Lørenskog, Norway; 3 Department of Tumour Biology, Oslo University Hospital, Oslo, Norway; 4 Institute of Clinical Medicine, University of Oslo, Oslo, Norway; 5 Department of Gastroenterological Surgery, Oslo University Hospital, Oslo, Norway; 6 Department of Oncology, Oslo University Hospital, Oslo, Norway; 7 Department of Radiology, Akershus University Hospital, Lørenskog, Norway; 8 Department of Pathology, Akershus University Hospital, Lørenskog, Norway; 9 Department of Oncology, Herlev and Gentofte Hospital, Herlev, Denmark; 10 Department of Physics, Norwegian University of Science and Technology, Trondheim, Norway

**Keywords:** Tumour-necrosis factors, Immunosurveillance, Rectal cancer, Prognostic markers, Predictive markers

## Abstract

**Background:**

In colorectal cancer, the inflamed tumour microenvironment with its angiogenic activities is immune- tolerant and incites progression to liver metastasis. We hypothesised that angiogenic and inflammatory factors in serum samples from patients with non-metastatic rectal cancer could inform on liver metastasis risk.

**Methods:**

We measured 84 angiogenic and inflammatory markers in serum sampled at the time of diagnosis within the population-based cohort of 122 stage I–III patients. In a stepwise manner, the statistically strongest proteins associated with time to development of liver metastasis were analysed in the corresponding serum samples from 273 stage II–III rectal cancer patients in three independent cohorts.

**Results:**

We identified the soluble form of the costimulatory immune checkpoint receptor cluster of differentiation molecule 40 (sCD40) as a marker of liver metastasis risk across all patient cohorts—the higher the sCD40 level, the shorter time to liver metastasis. In patients receiving neoadjuvant treatment, the sCD40 value remained an independent variable associated with progression to liver metastasis along with the local treatment response. Of note, serum sCD40 was not associated with progression to lung metastasis.

**Conclusions:**

Circulating sCD40 is a marker of liver metastasis risk in rectal cancer and may be developed for use in clinical practice.

## Background

Colorectal cancer (CRC) is a common malignancy with a sharp rise in incidence from the age of 60.^1^ Despite improved understanding of the biological complexity^2,3^ and increasingly individualised treatments based on biological characteristics,^4,5^ progression to liver metastasis remains an important cause of severe morbidity and dismal survival. The consensus molecular subtype classification of primary CRC tumours has provided a theoretical framework for the role of the immune system, which in a likely simplified manner, defines subtype-1 tumours as highly immunogenic and the subtype-4 counterpart with a chronically inflamed tumour microenvironment (TME) as immune-tolerant.^2^ In clinical practice, detrimental systemic inflammation can be regarded as a driver event for poor outcome of advanced CRC.^6^

Tumour-driven inflammation locally induces proangiogenic factors, which subsequently facilitate the migration of inflammatory cells into the TME^7,8^ and support suppressive cellular and signalling immune responses,^9^ thereby creating a vicious cycle favourable for tumour progression.^10^ In the liver, hepatocytes in concert with the inflammatory response incite the formation of pro-metastatic niches, paving the way for metastatic colonisation.^11^ Interestingly, perioperative chemotherapy containing the angiogenic inhibitor bevacizumab resulted in significantly better tumour response and survival outcomes for patients with resectable colorectal liver metastases with an angiogenic growth pattern within the liver tissue compared to cases devoid of this histologic feature.^12^ Recognising the functional network of interrelated angiogenic and immune-modulatory factors as relevant for liver tumours in particular, recent studies have explored the therapeutic efficacy of combining angiogenic inhibition with immune checkpoint blockade (ICB).^9,13^ In the clinical context, multiplex protein analysis of blood is a rational approach to search for mechanistically involved factors that may be candidates for new therapies. Conceptually, simultaneous assessment of a high number of circulating proteins can unveil the systemic manifestations of the TME biology as well as the constitutional and acquired physiology of the patient for this purpose.^14^

Rectal cancer comprises about a third of all CRC cases. When rectal cancer presents with extensive growth or lymph node involvement within the pelvic cavity, neoadjuvant treatment is given to reduce the risk of local recurrence after the pelvic surgery, but metastatic progression, especially to the liver, remains a common failure. Low primary rectal tumours have the propensity to metastasise to the lungs as the primary site,^15^ but patients with single-organ lung metastases have significantly better prognosis compared to those with other metastatic sites.^16^ Hence, to explore our hypothesis that angiogenic and inflammatory activity originating in the primary tumour and manifested systemically may impact on the risk of developing liver metastases in particular and potentially be targeted within an intensified multimodal therapy course, we measured 84 serum proteins (Supplementary Table S1) in specimens sampled at the time of diagnosis in a cohort of stage I–III patients. This population-based Investigation Cohort (IC) comprised 122 T2–T4 rectal cancer cases treated according to standard clinical guidelines.^17^ Circulating proteins that were found associated with liver metastasis risk were further validated in three independent cohorts of stage II–III patients, Validation Cohort (VC) 1–3, each with unique characteristics that together with the IC represented the full biological range of this heterogeneous disease. The VC1 was used because 35.4% of the 79 cases had organ-invasive (T4) disease that is at particularly high risk of poor outcome.^18^ The VC2 was chosen because 49.6% of the 135 cases had low primary tumour (5 cm or less from the anal verge) with the specific risk of developing lung metastases^19^ and therefore might challenge our hypothesis. The VC3 was analysed because its 59 cases resembled those of VC1.

## Methods

### Patients and procedures

Three-hundred and ninety-five patients with confirmed non-metastatic rectal adenocarcinoma from four prospective studies, approved by the pertinent Ethics Committees and Institutional Review Boards, were included in this post hoc analysis. All patients had given written informed consent for study participation with ancillary investigations. The patient cohorts with demographic data have been detailed in full previously.^19^ Briefly, the IC (NCT01816607) consisted of 122 stage I–III patients with a mean age of 64 years, enrolled at Akershus University Hospital (Lørenskog, Norway) between October 28th, 2013 and December 17th, 2017, and was censored on January 2nd, 2020. The study was designed as a prospective biomarker study for patients enrolled according to unselected recruitment. The VC1 (NCT00278694) consisted of 79 stage II–III patients with a mean age of 57 years, enrolled at Oslo University Hospital (Oslo, Norway) and Akershus University Hospital between October 5th, 2005 and March 3rd, 2010, and was censored on February 22nd, 2017. The study patients received neoadjuvant short-course oxaliplatin-based chemotherapy followed by long-course chemoradiotherapy before the radical surgery. The VC2 (NCT02113384) consisted of 135 stage II–III patients with a mean age of 63 years, enrolled at Oslo University Hospital between September 13th, 2012 and March 31st, 2016, and was censored on August 24th, 2018. This study was designed as a prospective biomarker study for patients with locally advanced tumours who were eligible for standard long-course chemoradiotherapy before the surgery. The VC3 (NCT00964457) consisted of 59 stage II–III RC patients with a mean age of 66 years, enrolled at Herlev and Gentofte Hospital (Herlev, Denmark) between July 6th, 2009 and October 27th, 2014, and was censored on October 1st, 2016. The study patients received a similar intensified neoadjuvant treatment regimen to that administered to the VC1 patients. All patients in the present study had standard diagnostic workup and evaluation of response to neoadjuvant therapy (ypTN staging) in the surgical specimen, including scoring of histologic tumour regression grade (TRG) (Supplementary materials and patient procedures). Each cohort was scored according to the preferred TRG protocol^20–22^ at the study site; to enable comparison across the cohorts, all TRG results were converted to the same scale, where TRG1 comprised both complete and near-complete tumour regression, moderate tumour regression was set to TRG2 and minimal-to-no tumour regression received the score of TRG3, in consultation with an experienced specialist in pathology. Follow-up consisted of clinical examination and CT scanning at three months following surgery, then every six months for two years and finally every year until five years. The various treatment regimens and clinical and pathological features of the patients in the different cohorts are summarised in Supplementary Table S2. At the last date of censoring, the median follow-up time for all cohorts was 34 months [minimum (min) 0, maximum (max) 60]. At this time, 46 patients had developed liver metastasis and 38 patients had metastasis to the lungs. The collected data were quality-controlled by the first author before the present analyses.

### Analysis of circulating proteins

Serum was collected at the time of study enrolment before any treatment and stored at –80 °C until analysis. In the IC, VC1 and VC3 specimens, the simultaneous analysis of 84 proteins related to angiogenesis and inflammation was undertaken with the required number of customised Luminex® Multiplex Assays (R&D Systems, Minneapolis, MN, USA). In VC2, the soluble cluster of differentiation molecule 40 (sCD40) was measured by single-parameter immunoassays (R&D Systems, product number DCCD40). All analytes were measured in duplicate in both assays.

### Statistical considerations

Analyses were performed using the IBM SPSS Statistics for Mac version 26, GraphPad Prism version 8.4.2 or R version 4.0.0. The 84 serum proteins were analysed in a stepwise process. First, we used the Significance Analysis for Microarrays (SAM) software version 5.0 in the R workspace, which handles any missing data by imputation using the K-nearest-neighbour method,^23^ to select the most significant proteins in the IC. Herein, differences in protein levels according to the time to liver metastasis detection were identified by a set of Cox regression analyses, where each protein received a score based on its difference relative to the standard deviation of repeat measurements.^23^ Next, we identified the statistically strongest proteins associated with this specific outcome for the IC, VC1, VC2 and VC3 cohorts progressively in univariable Cox proportional hazard models in SPSS. Finally, the top serum protein, sCD40, was analysed against established prognostic markers in a multivariable Cox proportional hazard model. The results are presented as hazard ratio (HR) with a 95% confidence interval (95% CI). The Kruskal–Wallis test or Mann–Whitney *U* test was used to determine differences between groups, and Dunn’s pairwise comparison was used as post hoc test for multiple comparisons. Correlations were calculated by the Spearman’s rho test and *P* values were corrected using the Holm–Šidák method. Survival was calculated in each cohort separately from the date of study enrolment to the date of detected metastasis in the liver, lungs or other sites, death from any cause or end of follow-up at 5 years, whichever occurred first. Crude survival differences were assessed by the log-rank test and visualised by the Nelson–Aalen cumulative hazard method. All tests were two-sided and *P* values of less than 0.05 were considered statistically significant.

## Results

### Angiogenesis and inflammation serum proteins and liver metastasis

In the IC, serum levels of ANGPT2, sCD40, C–X–C motif chemokine ligand 2 (CXCL2), tumour necrosis factor superfamily member 10B (TNFRSF10B), fms-related receptor tyrosine 3 ligand (FLT3LG) and vascular endothelial growth factor A (VEGFA) showed strong positive association with the time to detection of liver metastasis (Table 1, factors ranged by the relative difference in protein level according to the outcome, *D*).^23^ None of the 84 cytokines was associated with the time to detection of metastasis in lungs or other sites in the SAM survival analysis (not shown). As further shown in Table 1, the false discovery rate (*Q*) was 0 for CXCL2, ANGPT2, sCD40 and TNFRSF10B but 7.73 (still within the accepted limit of 10)^23^ for FLT3LG and VEGFA, indicating that the results were likely not random. However, only CXCL2 and sCD40 remained significant in the SPSS univariable Cox regression analysis with regard to higher levels associated with increased risk of progression to liver metastasis. Furthermore, only sCD40 upheld the association with the time to liver metastasis detection in VC1, leaving sCD40 as the only remaining candidate for analysis in VC2. Finally, sCD40 was validated as a potent marker of liver metastasis risk in VC3. When comparing sCD40 with prognostic markers in clinical use (circulating carcinoembryonic antigen at the time of diagnosis and neoadjuvant treatment response parameters ypT and TRG of the surgical specimen), sCD40 and TRG remained independent variables associated with progression to liver metastasis (Table 2).

**Table 1:** Circulating proteins and the development of liver metastasis—the separate cohorts.

	SAM analysis		Univariable Cox regression analysis		
	*D*	*Q*	*N* (%)	HR (95% CI)^a^	*P*
*Investigation cohort*^b^					
CXCL2	2.14	0	121 (99.2)	1.001 (1.000–1.002)	0.011
ANGPT2	1.55	0	121 (99.2)	1.000 (1.000–1.000)	0.833
sCD40	1.51	0	121 (99.2)	1.004 (1.001–1.007)	0.007
TNFRSF10B	1.35	0	121 (99.2)	1.000 (0.998–1.002)	0.904
FLT3LG	0.95	7.73	116 (95.1)	0.996 (0.982–1.011)	0.618
VEGFA	0.88	7.73	101 (82.8)	1.000 (1.000–1.000)	0.849
*Validation cohort 1*					
CXCL2			79 (100)	1.001 (0.999–1.002)	0.249
sCD40			79 (100)	1.003 (1.000–1.006)	0.040
*Validation cohort 2*^c^					
sCD40			129 (95.6)	1.001 (1.000–1.002)	0.029
*Validation cohort 3*^d^					
sCD40			55 (93.2)	1.003 (1.000–1.006)	0.044

*ANGPT2* angiopoietin-2, *CI* confidence interval, *CXCL2* C–X-C motif chemokine ligand 2, *D* likelihood score, *FLT3LG* fms-related receptor tyrosine kinase 3 ligand, *HR* hazard ratio, *Q* false discovery rate, *SAM* Significance Analysis for Microarrays, *sCD40* soluble cluster of differentiation molecule 40, *TNFRSF10B* tumour necrosis factor superfamily member 10B, *VEGFA* vascular endothelial growth factor A.

^a^HR above 1 indicates enhanced probability for the event. Patients were omitted from the analyses because they developed lung metastasis before the first occurrence of liver metastasis: ^b^one ^c^and additional three patients without metastatic disease who were lost to follow-up.

^d^Additional two patients who died without metastatic disease.

**Table 2:** Prognostic factors and the development of liver metastasis—all cohorts.

	Univariable Cox regression analysis			Multivariable Cox regression analysis		
	*N* (%)^a^	HR (95%CI)^b^	*P*	*N* (%)^a^	HR (95%CI)^b^	*P*
sCD40	389 (98)	1.002 (1.001–1.002)	<0.001	233 (59)	1.001 (1.000–1.002)	0.006
CEA	326 (83)	0.999 (0.987–1.011)	0.892	233 (59)	0.988 (0.966–1.010)	0.280
TRG	286 (72)	1.844 (1.246–2.729)	0.002	233 (59)	1.781 (1.028–3.084)	0.039
ypT	286 (72)	1.838 (1.270–2.661)	0.001	233 (59)	1.522 (0.966–2.399)	0.070

*CEA* carcinoembryonic antigen, *CI* confidence interval, *HR* hazard ratio, *sCD40* soluble cluster of differentiation molecule 40, *TRG* tumour regression grade, *ypT* histologic tumour stage following neoadjuvant treatment.

^a^Patients who developed lung metastasis (six cases) before the first occurrence of liver metastasis were omitted from the analysis.

^b^HR above 1 indicates enhanced probability for the event.

**Fig. 1:**
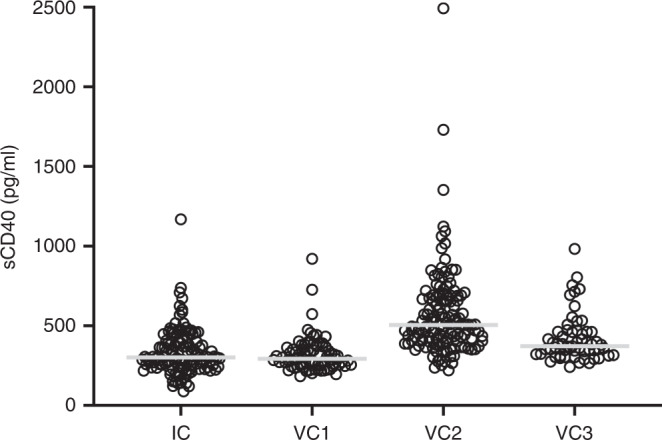
sCD40 serum leves in the different cohorts. Patients’ serum levels of the soluble cluster of differentiation molecule 40 (sCD40, open circles) in the Investigation Cohort (IC) and Validation Cohorts (VC1–3); grey line, the cohort median value.

**Fig. 2:**
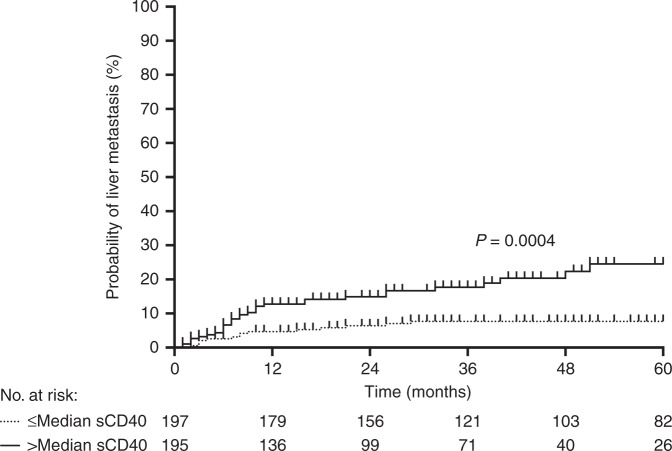
High or low sCD40 and risk of liver metastasis development. Cumulative percentages of cases with liver progression among all patients grouped with higher or lower than the median serum value of the soluble cluster of differentiation molecule 40 (sCD40).

The sCD40 levels as measured (in pg/ml, Fig. 1) with the multiplex assay batches displayed different levels across cohorts, with median values of 300.6 (min 88.10, max 1168) in IC, 291.9 (min 184.0, max 920.6) in VC1 and 372.2 (min 242.0, max 982.5) in VC3. The single-parameter analysis of VC2 resulted in a median sCD40 value of 504.9 (min 220.7, max 2493), which was significantly different (*P* < 0.001, by Kruskal–Wallis test) from the multi-parameter assay measures. When pooling all patients, those with serum sCD40 above the global median value of 371.3 had shorter time to development of liver metastasis (log-rank, *P* < 0.001, Fig. 2) with HR of 2.96 (95% CI, 1.57–5.56), *P* = 0.001. Serum sCD40 was not associated with progression to lung metastasis (Supplementary Fig. S1; HR 0.94 (95% CI, 0.49–1.82), *P* = 0.856). For distant metastasis-free survival, irrespective of metastatic organ, HR was 1.92 (95% CI, 1.31–2.83), *P* = 0.001 for the entire cohort. For overall survival, HR was 2.57 (95% CI, 1.70–3.89), *P* < 0.001.

In support of circulating sCD40 representing interrelated angiogenic and inflammatory responses in the patients (Table 3), we found weak correlations between sCD40 values and serum levels of other proteins identified by the SAM survival analysis (TNFRSF10B and FLT3LG), as well as the pro-inflammatory cytokine interleukin-6 (IL-6) that was also measured on the protein assay. Of note, a relatively strong correlation (rho of 0.501) was observed between increasing erythrocyte sedimentation rate and higher sCD40 value, altogether indicating an association with detrimental inflammatory activity.^24^ Declining haemoglobin and albumin in the circulation are markers of increasing severity of a malignant condition;^25^ we found an inverse but weak correlation between the levels of sCD40 and each of these factors. As the formation of liver pro-metastatic niches is dependent on functional hepatocytes,^11^ the sCD40 value showed weak correlation with circulating alanine aminotransferase as a marker of inflammatory damage of hepatocytes.^26^

**Table 3:** Correlations between the soluble cluster of differentiation molecule 40 and other circulating factors—all cohorts.

	*N* (%)	Spearman’s rho	*P*	*P*^a^
CXCL2	260 (66)	0.042	0.501	0.872
ANGPT2	395 (100)	0.083	0.100	0.403
TNFRSF10B	201 (51)	0.359	<0.001	<0.001
FLT3LG	245 (62)	0.345	<0.001	<0.001
VEGFA	180 (46)	–0.047	0.533	0.872
IL-6	164 (42)	0.296	<0.001	<0.001
TNF-α	177 (45)	0.125	0.098	0.403
IFN-γ	176 (45)	–0.052	0.496	0.872
Haemoglobin	334 (85)	–0.219	<0.001	<0.001
Thrombocytes	316 (80)	0.082	0.147	0.493
Neutrophils	300 (76)	0.093	0.107	0.493
Lymphocytes	298 (75)	–0.056	0.333	0.568
Monocytes	297 (75)	0.186	0.001	0.013
C-reactive protein	301 (76)	0.185	0.001	0.013
Albumin	315 (80)	–0.187	0.001	0.013
Carcinoembryonic antigen	333 (84)	0.163	0.003	0.027
Erythrocyte sedimentation rate	120 (30)	0.501	<0.001	<0.001
Creatinine	180 (46)	0.077	0.302	0.568
Aspartate aminotransferase	214 (54)	–0.139	0.042	0.291
Alanine aminotransferase	322 (82)	–0.235	<0.001	<0.001
Lactate dehydrogenase	283 (72)	0.193	0.001	0.013
Alkaline phosphatase	319 (81)	0.065	0.244	0.568
γ-glutamyl transferase	296 (75)	0.103	0.078	0.434
Bilirubin	318 (81)	–0.090	0.109	0.493

*ANGPT2* angiopoietin-2, *CXCL2* C–X-C motif chemokine ligand 2, *FLT3LG* fms-related receptor tyrosine kinase 3 ligand, *IFN-γ* interferon γ, *IL-6* interleukin-6, *TNF-α* tumour necrosis factor α, *TNFRSF10B* TNF superfamily member 10B, *VEGFA* vascular endothelial growth factor A.

^a^Corrected for multiple comparisons.

### Circulating sCD40, patients and the primary tumour

As summarised in Table 4, the serum sCD40 value was weakly correlated with the patient’s age but unrelated to female or male sex. Patients with stage II (T3–4N0) disease had significantly higher sCD40 levels than stage I and III patients, reflecting the highest sCD40 levels among T4 cases and lack of correlation with positive N stage. Post hoc analysis (Dunn’s pairwise comparison) confirmed the significant differences in sCD40 among disease stages, again with the highest values for stage II, as well as the significantly increasing sCD40 levels for more advanced T stages. The significantly lower sCD40 levels in patients who obtained TRG1 in the surgical specimen after neoadjuvant treatment, confirmed in the post hoc analysis with no difference between TRG2 and TRG3 cases, reflect the strong but independent association to the development of liver metastasis for sCD40 and TRG1, which was also apparent in the multivariable Cox regression analysis (Table 2).

**Table 4:** Associations between the circulating soluble cluster of differentiation molecule 40 and clinical parameters—all cohorts.

	*N* (%)	Median (min, max)	Spearman’s rho	*P*^a^
Age (years)	395 (100)	64 (30, 93)	0.237	<0.001
Sex				
Female	153 (39)	371.0 (88.1, 1730)		
Male	242 (61)	372.5 (120.1, 2493)		0.905
Stage				
I	30 (8)	283.1 (120.1, 624.5)		
II	106 (27)	448.8 (88.10, 2493)		
III	246 (62)	364.6 (151.8, 1730)		<0.001
X^b^	13 (3)	393.7 (148.0, 804.0)		
T				
2	37 (9)	286.7 (120.1, 624.5)		
3	185 (47)	362.3 (151.8, 2493)		
4	172 (44)	420.7 (88.10, 1353)		<0.001
X^b^	1 (0)	148.0		
N				
0	136 (34)	412.8 (88.10, 2493)		
1	98 (25)	363.5 (198.9, 1730)		
2	148 (37)	364.7 (151.8, 1017)		0.194
X^b^	13 (3)	393.7 (148.0, 804.0)		
ypT				
0	49 (17)	379.3 (88.10, 1122)		
1	19 (7)	301.6 (196.5, 616.1)		
2	52 (18)	383.8 (225.1, 852.6)		
3	127 (44)	365.0 (139.6, 2493)		
4	39 (14)	385.4 (224.4, 920.6)		0.058
ypN				
0	200 (70)	375.1 (88.10, 2493)		
1	61 (21)	352.9 (139.6, 1168)		
2	25 (9)	351.4 (151.8, 814.5)		0.470
pCR (ypT0N0)				
Yes	46 (16)	375.2 (88.10, 1122)		
No	240 (84)	370.3 (139.6, 2493)		0.830
TRG				
1	147 (51)	351.9 (88.10, 1122)		
2	68 (24)	430.1 (160.7, 2493)		
3	71 (25)	385.4 (139.6, 1093)		0.001

*max* maximum, min minimum, *pCR* pathological complete response, *TRG* tumour regression grade, *ypT* histologic staging following neoadjuvant treatment.

^a^By Spearman’s rho correlation (continuous variable) and Kruskal–Wallis test or Mann–Whitney *U* test, as appropriate (categorical variables).

^b^Unclassified cases are detailed in Supplementary materials and patient procedures.

## Discussion

We measured 84 circulating proteins associated with angiogenesis and inflammation in pre-treatment serum specimens sampled within a population-based study cohort of stage I–III rectal cancer patients and identified the soluble form of the CD40 costimulatory immune checkpoint receptor^27^ being a strong prognostic marker of the time to development of liver metastasis. The prognostic value was validated in three independent study cohorts of stage II–III rectal cancer patients characterised by a high percentage of cases with organ-invasive or low primary tumour, altogether representing the full biological range of this heterogeneous disease. Patients receiving neoadjuvant treatment with complete or near-complete treatment response of the surgical specimen had lower pre-treatment serum levels of sCD40, but the sCD40 value remained an independent variable associated with progression to liver metastasis along with the histopathological TRG1. Of note, the level of serum sCD40 was not associated with progression to lung metastasis. These observations may reflect the specific depletion of tumour-directed immune cells within the liver microenvironment.^28^

We hypothesised that the development of liver metastases is contingent on angiogenic and immune-modulatory activity driven by the progressing primary tumour and detectable in the circulation. We found that a high serum sCD40 level correlated with high erythrocyte sedimentation rate and decreased haemoglobin and serum albumin, suggesting a role of sCD40 in the interplay between systemic inflammation and an adverse disease course.^24^ The histopathological tumour response to neoadjuvant therapy is a strong determinant of long-term survival,^29,30^ herein the progression to liver metastasis.^19^ Since circulating sCD40, in contrast to the TRG parameter as the local response score, was available before any treatment started and also pertained to patients proceeding directly to pelvic surgery, it might be an ideal candidate for risk stratification and response prediction in rectal cancer, for instance in selecting patients for intensified neoadjuvant therapy^31^ or the addition of an immune-modulatory agent within the multimodal therapy course.

As a member of the tumour necrosis factor receptor superfamily, CD40 is a key regulator of adaptive immune responses.^32^ In its membrane-bound state (mCD40), it is found on the surface of several cell types, including endothelial cells,^33^ antigen-presenting dendritic cells^34^ and B cells;^35^ however, its expression shows low tissue specificity and has not been specifically detected in colon or rectum (www.proteinatlas.org).^36^ In advanced cancer, blockade of coinhibitory immune checkpoint proteins revokes evasion of T-cell cytotoxicity,^37–39^ a strategy adopted with great success in several cancer entities, including a small subgroup of highly immunogenic CRC.^40–42^ Most malignancies, however, are unresponsive to ICB,^43^ including the majority of CRC cases.^44^ Agonists of costimulatory immune checkpoint receptors have been advocated an alternative strategy to invoke antitumour immune activity, with antibodies targeting several proteins under development.^27^ In experimental models, agonistic CD40 antibodies have been explored in combination with anti-angiogenic agents^45^ or ICB and radiation.^46^ In an inherently immune-tolerant pancreatic cancer model, sequential radiotherapy, mCD40 activation and ICB led to an intratumoural shift of the T-cell populations from tumour-promoting to tumour-suppressing phenotypes, resulting in tumour eradication and long-term immunity.^46^ Another recent pancreatic cancer experiment supported these findings, demonstrating that restoration of functional dendritic cells in the TME by combining treatment with FLT3LG and agonistic mCD40 antibodies induced antitumour immunity and enhanced responsiveness to radiotherapy.^47^

However, the soluble form of CD40 comprises the extracellular domain of mCD40, generated via proteolytic cleavage from the surface of CD40-expressing cells.^48,49^ Thus, sCD40 can act as a natural antagonist of the mCD40–CD40 ligand (CD40L) interaction,^49^ implying that the shedding of sCD40 may represent a negative feedback control of mCD40-mediated functions. Early experimental studies showed that lack of CD40L or blocking of the mCD40–CD40L interaction led to severely impaired B-cell antibody production^50^ and thereby impaired adaptive immune responses.^51^ In a dose-escalating study with an agonistic CD40 antibody for patients with advanced solid tumours, half the participants at the maximum-tolerated dose experienced depletion of helper and cytotoxic T cells,^52^ suggesting that stimulation of the CD40–CD40L axis may have led to counterproductive immune modulation.^53^ Specifically, it has long been known that CD40–CD40L signalling in the interaction of various immune cell subsets enhances a counterbalancing apoptotic activity mediated by Fas–FasL.^54^ In this regard, recent experimental and patient data demonstrated that myeloid cells within colorectal liver metastases caused apoptotic depletion of tumour-directed T cells mediated via Fas–FasL signalling, which was not the case for colorectal lung metastases.^28^ We may speculate if circulating sCD40 reflects the liver specificity of disseminating CRC, as the organotropism is said to be largely based on the unique microenvironment, including the secretome, of the specific target organs for metastatic tumour cells.^55^ Measuring sCD40 in clinical CRC practice may be of note in this regard.

The patients of our four rectal cancer cohorts had been given diverse neoadjuvant treatment regimens and a fifth of the cases had proceeded directly to pelvic surgery. The data analysis was limited by the lack of systematic reporting of other known prognostic factors, for instance patient comorbidities and other tumour data, such as distance to the circumferential margin or the status of extramural vascular invasion. Moreover, the serum sCD40 measurement was undertaken with two different methods that displayed differences in measured sCD40 levels. Still, increasing levels of sCD40 remained significantly associated with shorter time to development of liver metastasis across all patient cohorts. It is tempting to speculate that high circulating sCD40 may have impeded a tumour-directed immune response protecting against metastatic colonisation of the liver and further suggests the determination of this immune-modulating factor to be integrated in the diagnostic workup for rectal cancer.

To our knowledge, we are the first to demonstrate that high circulating level of the sCD40 costimulatory immune checkpoint receptor is a marker of liver metastasis risk in CRC. Given this is validated in further studies, it should be feasible to use in clinical practice.

## Supplementary information

Supplementary Materials

## Data Availability

The datasets analysed during the current study are available from the corresponding author on reasonable request.
