# Supplementary material for: The circulating soluble form of the CD40 costimulatory immune checkpoint receptor and liver metastasis risk in rectal cancer
Source: Br J Cancer. 2021 Apr 9;125(2):240–6. doi: 10.1038/s41416-021-01377-y (PMC8292313; doi:10.1038/s41416-021-01377-y)
Supplement: Supplementary file 1 — Supplementary Materials [file 41416_2021_1377_MOESM1_ESM.docx]

**SUPPLEMENTARY MATERIALS**

**Patient procedures**

All patients had histologically confirmed non-metastatic rectal adenocarcinoma based on rectoscopy, pelvic MR imaging and thoracic/abdominal CT scan. Age and sex were available for all patients. The clinical TNM staging was undertaken at each study site without central review. One patient was diagnosed with adenocarcinoma in a rectal polyp that had been completely removed at a private institute a week before the study inclusion, making further clinical staging impossible. For 12 additional patients, the radiologists were unable to determine N stage. Standard clinical blood markers from the time of diagnosis were available with varying coverage for all cohorts except Validation Cohort 3. Of the 395 patients, 10 patients proceeded to chemotherapy only and 21 patients received radiotherapy for local control, in both instances pertaining to patients who were considered unresectable because of advanced age, comorbidity or other parameters related to the patient or cancer. Patients who received neoadjuvant treatment had either short-course or long-course radiotherapy with or without standard concomitant or sequential chemotherapy. Validation Cohorts 1 and 3 consisted of patients who had received study-specific oxaliplatin-containing induction chemotherapy and sequential chemoradiotherapy (Table S2). Two patients declined surgery after the neoadjuvant therapy due to personal opinion. Hence, a total of 362 cases underwent surgery, of whom 74 patients had direct surgery and 288 were resected after neoadjuvant therapy (Table S2). Of note, none of the patients received an angiogenic inhibitor as part of the neoadjuvant treatment. Histologic ypTN staging and tumour regression grade (TRG) of the surgical specimen were available for 286 patients with neoadjuvant treatment. The TRG was assessed according to the preferred protocol at the study site. Patients included in cohorts until 2010 were given TRG between 1 and 5, where TRG1 represented complete eradication of tumour cells and TRG5 no treatment effect.^1^ After 2010, complete response was given TRG0 and no treatment effect TRG3.^2^ The Validation Cohort 3 scores spanned from complete response at TRG1 to no treatment response at TRG4.^3^ To enable comparison across the cohorts, all TRG results were converted to the same scale (spanning from complete or near complete tumour regression at TRG1 to no regression at TRG3) by the first author in consultation with an experienced specialist in pathology. A statistical comparison of the different cohorts according to clinical and pathological features has been undertaken with Chi-square test (Table S2). Outcome of the study-specific oxaliplatin-containing induction chemotherapy given in addition to chemoradiotherapy in Validation Cohorts 1 and 3 was not compared to outcome of standard neoadjuvant treatment given to the patients in the remaining cohorts. This was due to the lack of randomisation between the treatment regimens and the clinical and pathological differences between the cohorts, irrespective of treatment choice.

**REFERENCES**

1 Bouzourene, H., Bosman, F. T., Seelentag, W., Matter, M. & Coucke, P. Importance of tumor regression assessment in predicting the outcome in patients with locally advanced rectal carcinoma who are treated with preoperative radiotherapy. *Cancer* **94**, 1121-1130 (2002).

2 Bateman, A. C., Jaynes, E. & Bateman, A. R. Rectal cancer staging post neoadjuvant therapy--how should the changes be assessed? *Histopathology* **54**, 713-721 (2009).

3 Mandard, A. M. *et al.* Pathologic assessment of tumor regression after preoperative chemoradiotherapy of esophageal carcinoma. Clinicopathologic correlations. *Cancer* **73**, 2680-2686 (1994).

**Table S1.** The 84 proteins analysed by the Luminex® Multiplex Assays (R&D Systems)

| AGER | CSF1 | CXCL14 | IL1R1 | PDGFB | TGFA |
| --- | --- | --- | --- | --- | --- |
| ANG | CSF2 | CXCL16 | IL2 | PDGFC | THBS2 |
| ANGPT1 | CSF3 | EGF | IL4 | PDGFD | TIE1 |
| ANGPT2 | CX3CL1 | ERBB2 | IL5 | PECAM1 | TIMP1 |
| CCL2 | CXCL1 | FAS | IL6 | PF4 | TNF |
| CCL3 | CXCL2 | FASLG | IL6ST | PLAUR | TNFRSF1A |
| CCL4 | CXCL5 | FGF2 | IL10 | PPBP | TNFRSF1B |
| CCL5 | CXCL6 | FLT3LG | IL12A | S100A8 | TNFRSF10B |
| CCL13 | CXCL8 | FLT4 | IL17A | SELE | TNFRSF10C |
| CCL17 | CXCL9 | HGF | LCN2 | SELL | TNFSF10 |
| CCL18 | CXCL10 | ICAM1 | MMP9 | SELP | TNFSF11 |
| CD27 | CXCL11 | IFNG | NCAM1 | SPARC | TPO |
| CD40 | CXCL12 | IL1A | PCT | SSP1 | VCAM1 |
| CD40LG | CXCL13 | IL1B | PDGFA | TEK | VEGFA |

Each protein is designated by the gene name according to the Human Genome Organization’s Gene Nomenclature Committee.

**Table S2.** The different patient cohorts – treatment regimens and clinicopathological features

|  |  | **IC** | **VC1** | **VC2** | **VC3** | **All cohorts** |  |
| --- | --- | --- | --- | --- | --- | --- | --- |
|  |  | *N* (%) | *N* (%) | *N* (%) | *N* (%) | *N* (%) | *P* |
| Sex | Female | 37 (30) | 34 (43) | 56 (41) | 26 (44) | 153 (39) |  |
|  | Male | 85 (70) | 45 (57) | 79 (59) | 33 (56) | 242 (61) | 0.146 |
| Surgery only | | 69 (57) | 0 (0) | 4 (3) | 1 (2) | 74 (19) |  |
| SCRT before surgery | | 6 (5) | 0 (0) | 7 (5) | 0 (0) | 13 (3) |  |
| LCRT before surgery* | | 41 (34) | 0 (0) | 103 (76) | 0 (0) | 144 (36) |  |
| Induction chemotherapy and sequential LCRT before surgery | | 0 (0) | 79 (100) | 0 (0) | 54 (91) | 133 (34) |  |
| SCRT or LCRT only | | 2 (2) | 0 (0) | 15 (11) | 4 (7) | 21 (5) |  |
| Chemotherapy only | | 4 (3) | 0 (0) | 6 (5) | 0 (0) | 10 (3) |  |
| Tumour location (cm from anal verge) | Low (<5 cm) | 41 (34) | 34 (43) | 67 (50) | 12 (20) | 154 (39) |  |
|  | Middle (5-10 cm) | 42 (34) | 31 (39) | 41 (30) | 25 (42) | 139 (35) |  |
|  | High (>10 cm) | 35 (29) | 14 (18) | 27 (20) | 22 (37) | 98 (25) | 0.003 |
|  | X† | 4 (3) | 0 (0) | 0 (0) | 0 (0) | 4 (1) |  |
| T stage | 1-2 | 31 (25) | 5 (6) | 1 (1) | 0 (0) | 37 (9) |  |
|  | 3 | 58 (48) | 46 (59) | 52 (39) | 28 (47) | 185 (46) |  |
|  | 4 | 31 (25) | 28 (35) | 82 (60) | 31 (53) | 172 (44) | <0.001 |
|  | X† | 1 (1) | 0 (0) | 0 (0) | 0 (0) | 1 (0) |  |
| N stage | 0 | 69 (57) | 9 (11) | 43 (32) | 15 (25) | 136 (34) |  |
|  | 1 | 40 (33) | 9 (11) | 35 (26) | 14 (24) | 98 (25) |  |
|  | 2 | 12 (10) | 60 (76) | 57 (42) | 19 (32) | 148 (37) | <0.001 |
|  | X† | 1 (1) | 1 (1) | 0 (0) | 11 (19) | 13 (3) |  |
| Stage | I | 28 (23) | 2 (3) | 0 (0) | 0 (0) | 30 (8) |  |
|  | II | 41 (34) | 7 (9) | 43 (32) | 15 (25) | 106 (27) |  |
|  | III | 52 (43) | 69 (87) | 92 (68) | 33 (56) | 246 (62) | <0.001 |
|  | X† | 1 (1) | 1 (1) | 0 (0) | 11 (19) | 13 (3) |  |
| ypT stage | 0 | 8 (17) | 16 (20) | 20 (18) | 5 (9) | 49 (17) |  |
|  | 1 | 5 (11) | 8 (10) | 3 (3) | 3 (6) | 19 (7) |  |
|  | 2 | 6 (13) | 17 (22) | 23 (21) | 6 (11) | 52 (18) |  |
|  | 3 | 25 (53) | 22 (28) | 52 (47) | 28 (52) | 127 (44) |  |
|  | 4 | 3 (6) | 15 (19) | 11 (10) | 10 (19) | 39 (14) | 0.026 |
|  | X† | 0 (0) | 1 (1) | 1 (1) | 2 (4) | 4 (1) |  |
| ypN stage | 0 | 29 (62) | 53 (67) | 77 (70) | 41 (76) | 200 (70) |  |
|  | 1 | 12 (26) | 20 (25) | 21 (19) | 8 (15) | 61 (21) |  |
|  | 2 | 6 (13) | 5(6) | 11 (10) | 3 (6) | 25 (9) | 0.499 |
|  | X† | 0 (0) | 1 (1) | 1 (1) | 2 (4) | 4 (1) |  |
| TRG | 1 | 19 (40) | 57 (72) | 51 (46) | 20 (37) | 147 (51) |  |
|  | 2 | 20 (43) | 12 (15) | 36 (33) | 0 (0) | 68 (24) |  |
|  | 3 | 8 (17) | 9 (11) | 22 (20) | 32 (59) | 71 (25) | <0.001 |
|  | X† | 0 (0) | 1 (1) | 1 (1) | 2 (4) | 4 (1) |  |
| pCR (ypT0N0) | Yes | 7 (15) | 15 (19) | 19 (17) | 5 (9) | 46 (16) |  |
|  | No | 40 (85) | 63 (80) | 90 (82) | 47 (87) | 240 (84) | 0.497 |
|  | X† | 0 (0) | 1 (1) | 1 (1) | 2 (4) | 4 (1) |  |

Cohorts are compared using Chi-square test, and *P* values are given for differences between the cohorts.

*Two patients declined surgery after the neoadjuvant therapy due to personal opinion. † Missing values. IC, Investigation Cohort; LCRT, long-course radiotherapy: 50 Gy delivered in 25 fractions over 5 weeks with or without concomitant chemotherapy; pCR, pathological complete response; SCRT, short-course radiotherapy: 25 Gy delivered in 5 fractions over 1 week; VC, Validation Cohort.

**Fig. S1**

Cumulative percentages of cases with lung progression among all patients grouped with higher or lower than the median serum value of the soluble cluster of differentiation molecule 40 (sCD40).
